# Vitamin C to Improve Organ Dysfunction in Cardiac Surgery Patients—Review and Pragmatic Approach

**DOI:** 10.3390/nu10080974

**Published:** 2018-07-27

**Authors:** Aileen Hill, Sebastian Wendt, Carina Benstoem, Christina Neubauer, Patrick Meybohm, Pascal Langlois, Neill KJ Adhikari, Daren K. Heyland, Christian Stoppe

**Affiliations:** 1 Department of Intensive Care Medicine, University Hospital RWTH, D-52074 Aachen, Germany; cbenstoem@ukaachen.de (C.B.); cneubauer@ukaachen.de (C.N.); 2 Department of Anesthesiology, University Hospital RWTH, D-52074 Aachen, Germany; 3 3CARE—Cardiovascular Critical Care & Anesthesia Evaluation and Research, D-52074 Aachen, Germany; swendt@ukaachen.de; 4 Department of Thoracic, Cardiac and Vascular Surgery, University Hospital RWTH, D-52074 Aachen, Germany; 5 Department of Anesthesiology and Intensive Care, University Hospital Frankfurt, D-60590 Frankfurt, Germany; patrick.meybohm@kgu.de; 6 Department of Anesthesiology and Reanimation, Faculty of Médecine and Health Sciences, Sherbrooke University Hospital, Sherbrooke, Québec, QC J1H 5N4, Canada; Pascal.laferriere-langlois@usherbrooke.ca; 7 Department of Critical Care Medicine, Sunnybrook Health Sciences Centre, Interdepartmental Division of Critical Care Medicine, University of Toronto; Toronto, ON M4N 3M5, Canada; neill.adhikari@sunnybrook.ca; 8 Clinical Evaluation Research Unit, Kingston General Hospital, Kingston, ON K7L 2V7, Canada; dkh2@queensu.ca

**Keywords:** vitamin C, ascorbic acid, cardiac surgery, antioxidant therapy, nutrient, oxidative stress, organ dysfunction, multi organ failure

## Abstract

The pleiotropic biochemical and antioxidant functions of vitamin C have sparked recent interest in its application in intensive care. Vitamin C protects important organ systems (cardiovascular, neurologic and renal systems) during inflammation and oxidative stress. It also influences coagulation and inflammation; its application might prevent organ damage. The current evidence of vitamin C’s effect on pathophysiological reactions during various acute stress events (such as sepsis, shock, trauma, burn and ischemia-reperfusion injury) questions whether the application of vitamin C might be especially beneficial for cardiac surgery patients who are routinely exposed to ischemia/reperfusion and subsequent inflammation, systematically affecting different organ systems. This review covers current knowledge about the role of vitamin C in cardiac surgery patients with focus on its influence on organ dysfunctions. The relationships between vitamin C and clinical health outcomes are reviewed with special emphasis on its application in cardiac surgery. Additionally, this review pragmatically discusses evidence on the administration of vitamin C in every day clinical practice, tackling the issues of safety, monitoring, dosage, and appropriate application strategy.

## 1. Introduction

### 1.1. Pathogenesis of Organ Dysfunction after Cardiac Surgery

**Figure 1:**
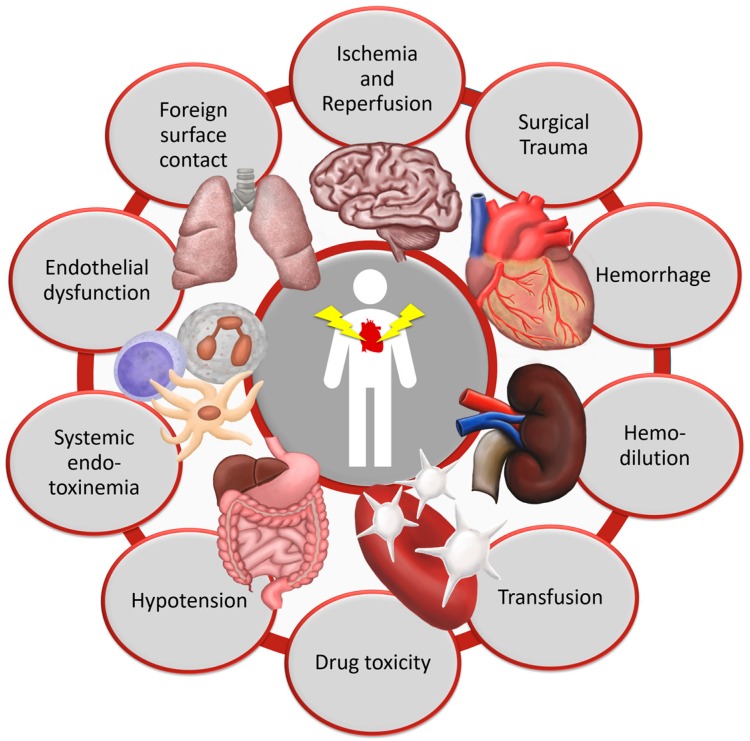
Pathophysiological mechanisms of organ damage in cardiac surgery.

Patients undergoing cardiac surgery experience a complex systemic inflammatory response syndrome (SIRS). SIRS after cardiac surgery is induced by surgical trauma [1,2], foreign surface contact during cardiopulmonary bypass (CPB) [2,3,4,5,6,7,8,9], CPB itself [1,9,10,11,12], ischemia-reperfusion-injury (I/R) [3,10,13], endotoxemia [3,10] and blood transfusion [10,14,15], as shown in Figure 1. Each stimulus triggers both the cellular and the humoral inflammatory response systems. Cellular mechanisms include the activation of leukocytes, platelets and endothelial cells [2,3,10,12,13]. Humoral reactions are mainly the activation of complement and coagulation systems, as well as the release of inflammatory mediators and reactive oxygen species [2,5,10,13].

Oxidative stress is defined as an imbalance between production of oxidants, mainly free radicals and reactive metabolites, in relation to their elimination by protective mechanisms. In many acute stages of disease, the production of reactive oxygen species (ROS) is initiated by several conditions, for example I/R-injury, activation of the Nicotinamide Adenine Dinucleotide Phosphate (NADPH) oxidase, as well as severe alterations in the mitochondrial metabolism [16]. ROS play an essential role in human biology and regulate different metabolic processes and signaling pathways. In critical illness, such as trauma, surgery, ischemia and reperfusion, shock and sepsis, the ROS production increases and often exceeds the natural antioxidant capacity, leading to damage of macromolecule structures. Structural damage of macromolecules such as proteins, nucleic acids, lipids and carbohydrates impairs their essential biological function and leads to significant damage of cell structure and organ function [17]. General activation of the inflammatory system and oxidative stress lead to leukocyte extravasation, intravascular leukostasis, lipid peroxidation, cell death, vasodilation and capillary fluid leakage in tissues, which negatively influence patient outcome [13,18,19,20,21,22]. 

While SIRS is a well-known reaction to cardiac surgery, it can cause multiple acute and persistent organ dysfunctions, which are explained in greater detail in Section 3. Postoperative complications, especially organ failure and infections, are major determinants of morbidity and mortality, necessitating prolonged hospital and intensive care unit (ICU) length-of-stay (LOS), which is further associated with high care-related costs and worse quality of life after cardiac surgery [2,3,10,11,23,24,25]. In fact, the development of acute and persistent multi-organ dysfunction occurs in 15% of patients and is the most important determinant of mortality, clinical outcome and quality of life for patients who underwent cardiac surgery [23,25].

### 1.2. Basic Metabolism and Functions of Vitamin C

Vitamin C is an essential micronutrient involved in numerous biochemical and biological processes. Two forms of vitamin C are present in plasma: Ascorbic acid (AA) and its oxidized form dehydroascorbate (DHA). The latter contributes less than 10% to the total ascorbate in human plasma [26]. The human body is unable to synthesize vitamin C due to lack of the last enzyme in the biosynthetic process. An adequate intake of vitamin C of 200 mg/day, equaling approximately five servings of fruit and vegetables is recommended, though food content varies due to its lability [27]. Vitamin C is absorbed enterally, remains unbound in human plasma, and is dialyzable. Renal elimination of vitamin C follows its glomerular filtration, if the concentration of vitamin C in the urine is larger than the capacity of the responsible transport protein, which is achieved by vitamin C uptake of 100 mg/day and a plasma concentration of 60 µmol/L [27].

Almost complete bioavailability was calculated in several models for dosages of 200 mg/day. Levine et al. observed tissue saturation of vitamin C occurring at intakes of 100 mg/day in adult healthy adult men [27,28]. Tissue saturation was assessed by vitamin C concentrations in lymphocytes, monocytes, and neutrophils. A steep sigmoidal relationship between vitamin C dose and steady-state plasma concentration was observed, where a dose of 200 mg produced approximately 80% plasma saturation, while plasma saturation occurred at about 1000 mg of vitamin C. However, the saturation of cells occurs at 100 mg/day due to active vitamin C transport, which saturates at about 60–70 µmol/L. The peak plasma concentration is reached about 2 h after ingestion, while an exponential drop of plasma levels is observed after intravenous application of vitamin C, where a half-life of vitamin C in plasma of approximately one hour was observed [27].

Vitamin C acts as an electron donor and has pleiotropic functions in the human body, being required by more than 60 enzymes. Among these, vitamin C dependent reactions are the synthesis of norepinephrine, collagen and carnitine. Furthermore, vitamin C dependent mono- and dioxygenases are involved in peptide amidation and tyrosine metabolism [29,30]. In addition, vitamin C plays a pivotal role in the metabolism of cholesterol to bile acids and in steroid metabolism [29,30]. Besides, vitamin C is known to support the cytochrome P450 driven hydroxylation or aromatic drugs and carcinogens [30] and to promote iron absorption in the small intestine [27].

Vitamin C enhances cell differentiation from somatic cells to induced pluripotent stem cells [31,32], or from stem cells to cardiomyocytes [33,34], which may be an important feature during various regenerating processes in chronically or acute critically ill patients. Previous studies have demonstrated that vitamin C also acts on epigenetic mechanisms [35]. Yet, given the limited data available in the setting of acute critical illness, studies are encouraged to explore this in the near future.

Based on its redox-potential and powerful antioxidant capacity, vitamin C has been called the most important antioxidant that counters the influence of free radicals [36,37]. The most relevant biochemical pathways concerning the development of organ dysfunctions are illustrated for each individual organ system in Section 2. Although important, a more comprehensive description of the underlying biochemical mechanisms and the influences of vitamin C are explained in greater detail elsewhere [29,30,38,39,40].

### 1.3. The Influence of Vitamin C on Oxidative Stress and Inflammation

Vitamin C scavenges free radicals through the formation of ascorbyl radical and thereby prevents damage to macromolecules such as lipids or the DNA. The dismutation of two ascorbyl radicals produces one molecule of ascorbate and one molecule of DHA [41]. Additionally, vitamin C inhibits the expression of intracellular adhesion molecules and thereby inhibits the intake of immune cells into the microcirculation [41]. Furthermore, an increase of the intracellular vitamin C concentration inhibits the protein phosphatase type 2A and thereby protects the endothelial barrier from septic shock [42]. Due to its pleiotropic functions in eight enzymatic processes, vitamin C not only mitigates oxidative stress, but also restores vascular responsiveness to vasoconstrictors [43], ameliorates microcirculatory blood flow, preserves endothelial barriers [40], prevents apoptosis [44] and augments bacterial defense [40]. 

### 1.4. Current Evidence of Vitamin C in Critically Ill Patients

Sepsis, trauma, burn and surgery are causes of systemic inflammatory responses and can lead to similar pathologies in the human body, including microvascular dysfunction, refractory vasodilatation, endothelial barrier dysfunction, edema and disseminated intravascular coagulation [45]. Vitamin C concentrations are lowered in critical illness [46], in patients recovering from surgery [47,48], in patients after cardiac surgery [49] and especially in patients heading towards multi-organ failure [19,50]. Fowler et al observed a lower rate of organ dysfunction as assessed by the sequential organ failure assessment (SOFA) score and a reduced 28-day mortality after the application of vitamin C in patients with sepsis and multi-organ-failure, whereas an influence on the ICU-LOS was not observed [51]. Zabet et al. demonstrated in 2016 patients a significantly reduced mean vasopressor demand and shorter duration of vasopressor therapy and reduced mortality in 28 septic patients receiving vitamin C [52]. In 2002, Nathens et al. observed a decreased risk of pneumonia, acute respiratory distress syndrome (ARDS) and a tendency towards lower alveolar inflammation in a randomized controlled trial (RCT) of antioxidant supplementation (1 g vitamin C and 1.000 IU vitamin E intravenously three times per day for up to 28 days) in mostly trauma patients (*n* = 595), although the results of this RCT did not reach statistical significance [53]. In severe burn patients, ascorbic acid reduced fluid demand and increased urine production, in a retrospective review by Kahn et al. [54] and in an RCT by Tanaka et al. [55]. In fact, the application of vitamin C is frequently considered in the treatment of severe burn patients [56]. While an overview of the influence of vitamin C on organ dysfunction is summarized in Table 1, Section 2 will take a closer look at each individual organ system.

## 2. Influence of Vitamin C on Organ Systems in Cardiac Surgery Patients

### 2.1. Nervous System

#### 2.1.1. Neuropsychological Dysfunction after Cardiac Surgery

**Figure 2:**
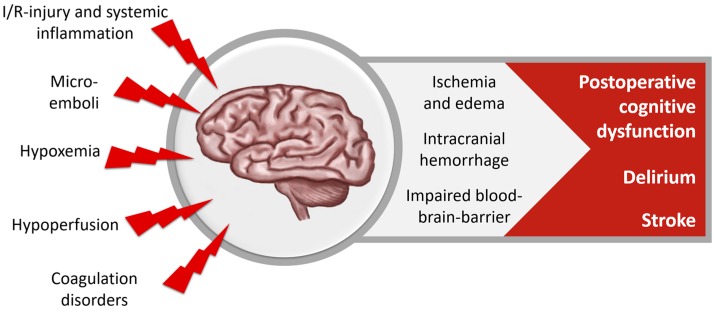
Cerebral dysfunction after cardiac surgery.

Brain tissue is very susceptible to oxidative damage because of its high content of polyunsaturated fatty acids and its high demand for oxygen. Neuropsychological complications are commonly seen in patients undergoing cardiac surgery, leading to a prolonged ICU stay (Figure 2). The American College of Cardiology and the American Heart Association defined two classes of neurological complications after cardiac surgery: Type I neurological deficits include stroke and transient ischemic attack, coma and fatal cerebral injury, Type II include delirium and postoperative cognitive dysfunction [65]. 

Cerebral ischemia due to stroke, microembolization, hypoperfusion, or hypoxemia contributes considerably to cognitive impairment. New cerebral lesions occur in about 30–50% of cardiac surgery patients, but most of them are clinically inapparent. The incidence of manifest stroke with clinical deficits is about 1–2% after low-risk heart surgery [66,67,68,69,70]. Contributing factors are major bleeding and transfusions of red blood cells, preoperative use of unfractionated heparin, and use of CPB [66]. Delirium is observed in a quarter and postoperative cognitive dysfunction is observed in 25–65% of all patients, while most of these patients recover within the first months [67,71]. Cognitive function is strongly influenced by systemic inflammation reaction, leading to increased permeability of the blood-brain barrier and cerebral edema [3]. All neuropsychological complications are associated with decreased quality of life, inability to work, loss of independence, and increased mortality [70].

#### 2.1.2. Role of Vitamin C in the Nervous System

Vitamin C levels are elevated up to 80 times in the cells of the brain and up to four times in the cerebrospinal fluid, compared to plasma, due to its active transport via the sodium-dependent vitamin C transporter-2 (SVCT2) transporter [72,73], protecting neurons and leukocytes from oxidative damage [49]. Vitamin C is also essential for the myelination of the neurons [74] and a vitamin C deficiency through insufficient transporter molecules leads to hypomyelination and collagen-containing extracellular matrix deficits [72]. If oxidized, vitamin C can also be taken up by glucose transporters [73]. During I/R injury or stroke, the vitamin C is shifted from the intracellular to the extracellular compartment, leading to an intracellular vitamin C deficiency and perhaps neuronal damage [49]. 

While there is evidence that vitamin C reduces infarct volume in cerebral ischemia, most evidence is derived from experimental studies inducing stroke or I/R-injury; reduced infarct volumes after experimental stroke models were demonstrated by Henry et al. [75] and Huang et al. [58]. This finding was supported by a recent study demonstrating that vitamin C protects from neuronal cell death in a model of ethanol-induced damage in early development age [57]. Ethanol thereby induced the development of oxidative stress. Amongst others, the protection was evaluated by reduced activation of caspase-9 and 3 as well as reduced levels of cytochrome C [57]. Lagowska-Lenard et al. found elevated antioxidant levels in the serum after vitamin C supplementation in a placebo-controlled RCT in patients with ischemic stroke. However, in this small study, the clinical outcome was unchanged [76].

#### 2.1.3. Vitamin C’s Influence on the Nervous System in Cardiac Surgery Patients

In the meta-analysis of Hu et al. 2017 including eight RCTs and 1060 patients, vitamin C supplementation had no effect on the incidence of stroke (0.8% (vitamin C) vs. 2.0 % (control)) in cardiac surgery patients [59]. To our knowledge, until now, no study evaluated the influence of vitamin C on cognitive dysfunction or delirium in cardiac surgery patients.

### 2.2. Cardiovascular System

#### 2.2.1. Cardiovascular Dysfunction after Cardiac Surgery

**Figure 3:**
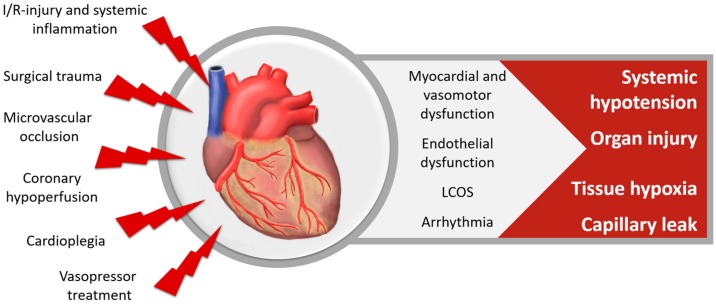
Cardiovascular dysfunction after cardiac surgery, LCOS = low cardiac output syndrome.

Surgical trauma, myocardial I/R, the excretion of inflammatory mediators, intraoperative cardioplegic arrest, reduced coronary blood flow and microvascular occlusion lead to a decline of myocardial contractility and a reduction of ventricular compliance and resulting function, as displayed in Figure 3. Vasodilation and decreased systemic vascular resistance contribute to systemic hypotension as well. Therefore, vasopressor treatment is commonly needed to support the circulation perioperatively in cardiac surgery patients. While vasopressor treatment is required to maintain adequate blood pressures, its use is associated with increased oxidative stress, endothelial dysfunction and myocardial fibrosis [77].

Myocardial dysfunction and cardiovascular insufficiency after cardiac surgery can cause a mismatch of oxygen delivery and metabolic demand and lead to tissue hypoxia. Ventricular systolic and diastolic dysfunction occurs in up to 70% of cardiac surgery patients [78,79]. The low cardiac output syndrome is clinically characterized by hypotension and signs of tissue hypoperfusion and occurs in 5–15% after cardiac surgery [78,80]. Acute kidney injury (AKI) as well as neurologic and pulmonary complications are the most common consequences of low cardiac output syndrome, leading to a mortality rate of more than 20% [3,79,81]. Arrhythmias are very common after cardiac surgery. Their impact on the clinical outcome depends on the kind of arrhythmia, its duration, ventricular response rate and cardiac function [82]. Arrhythmias might be I/R- and inflammation-induced and result from an increased intracellular calcium concentration due to calcium-influx through the damaged, peroxided lipids in the cell membranes, as well as hindered calcium uptake by the sarcoplasmic reticulum [49].

#### 2.2.2. Role of Vitamin C in the Cardiovascular System

Vitamin C affects the cardiovascular system through several pathways. Despite its capability to scavenge free radicals, vitamin C also promotes the differentiation of embryonic and pluripotent stem cells into cardiac myocytes [33,34]. Vitamin C has cardioprotective properties, which were demonstrated in rat models, where vitamin C reduced oxidative damage in diabetic rats [83] and during I/R-injury [84]. Vitamin C improved myocardial stunning and increased left ventricular function in some animal studies, however, other animal studies showed no effect of vitamin C and some only in combination with other antioxidants [49]. Therefore, preclinical data regarding the myocardial protection through vitamin C in I/R-injury remains inconclusive, as discussed in detail in a review by Spoelstra-de Man et al. [49].

Vitamin C inhibits the expression of inducible nitric oxide synthetase (iNOS) in endothelial cells and neuronal nitric oxide synthetase (nNOS) and thereby lowers the plasmatic level of nitric oxide (NO), which is responsible for the activation of guanylate cyclase that counteracts the effects of vasoconstrictors. Vitamin C also prevents the impairment of vasoconstriction [43] and restores inter-endothelial electrical coupling through connexin 37-contaning gap-junctions as well as through protein kinase A-activation required for connexin 40 dephosphorylation [43]. Therefore, vitamin C might increase vasopressor-sensitivity. However, in patients with endothelial dysfunction due to cardio-metabolic diseases, such as hypertension, atherosclerosis, diabetes and smokers, vitamin C promotes endothelial- and nitric oxide-dependent vasodilation [61]. Overall, vitamin C might improve micro-perfusion [43,47]. 

In extension, ascorbate also tightens the endothelial permeability barrier [61] and thus might lead to reduced extravasation and edema [85]. A meta-analysis including 44 RCTs and 1129 patients displayed an overall positive effect of vitamin C on endothelial function independently of baseline plasma concentration or route of administration [62]. In the studies included in this meta-analysis, endothelial function was assessed using ultrasound, plethysmography and pulse wave analysis. The effects were significant in patients with cardio-metabolic disorders, especially with heart failure (*p* < 0.02), atherosclerosis (*p* < 0.001) and diabetes (*p* < 0.001).

#### 2.2.3. Vitamin C’s Influence on the Cardiovascular System in Cardiac Surgery Patients

In cardiac surgery with CPB, vitamin C levels decrease with the production of ROS and remain low for days after surgery [49], indicating a greater demand of vitamin C in the setting of surgery and I/R-induced oxidative stress. Oxidative stress and myocardial damage after cardiac surgery with CPB might be decreased by the administration of vitamin C, as demonstrated in an RCT by Dingchao et al. in the 1990s [86]. In this RCT including 85 patients, the intervention group received a total of 250 mg/kg vitamin C before and after CPB. Markers for myocardial injury creatine kinase (CK) and creatine phosphokinase isoenzyme muscle/brain (CK-MB), as well as malondialdehyde as a marker for oxidative stress were significantly lower in patients receiving vitamin C. Clinically, the cardiac index was higher, and the intervention-group patients were less likely to need defibrillation after weaning from cardiopulmonary bypass and had shorter ICU- and hospital-LOS [86].

Vitamin C treatment also improves ventricular function, reduces vasopressor and fluid demand [86,87] and increases the cardiac index. In a systematic review [88] and in 6 different meta-analyses including 8–15 RCTs [59,60,63,89,90,91], vitamin C was shown to significantly reduce the occurrence of postoperative cardiac arrhythmia, mainly atrial fibrillation (AF). However, the results of these meta-analyses might be strongly influenced by publication bias, as discussed by Hemilae [92]. While postoperative AF gained increasing attention over the past years, and was investigated by several RCTs and meta-analyses, to our knowledge, no large, multicenter study evaluated the effect of vitamin C on other important outcomes, such as myocardial function or vasopressor and fluid-demand.

### 2.3. Respiratory System

#### 2.3.1. Pulmonary Dysfunction after Cardiac Surgery

**Figure 4:**
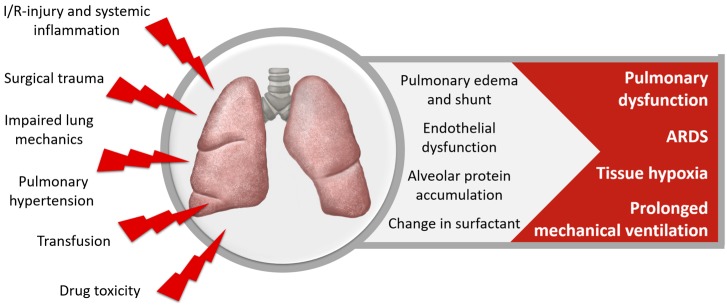
Pulmonary dysfunction after cardiac surgery.

Pulmonary dysfunction (Figure 4) occurs in up to 79% of patients after cardiac surgery, ranging from mild subclinical functional changes to acute respiratory distress syndrome (ARDS) in less than 2% of patients [93]. Acute lung injury is characterized by inflammation, and tissue damage is dealt mainly through oxidative stress and free radicals [94]. ROS like nitric oxide and superoxide can nitrate and oxidize key amino acids in lung proteins, such as surfactant protein, disturbing their function [95]. 

Factors contributing to pulmonary dysfunction are poor lung mechanics, increased intrapulmonary shunt and vascular resistance, pulmonary edema, changes in surfactant and alveolar protein accumulation. The underlying pathomechanisms include inflammation and free radicals, I/R-injury, transfusion-associated lung injury and drug toxicity. Pulmonary dysfunction causes prolonged need for mechanical ventilation, increases ICU- and hospital-LOS and mortality, and significantly affects long-term physical and psychological morbidity [3,96,97,98,99,100].

#### 2.3.2. Role of Vitamin C in the Respiratory System

Vitamin C functions as an antioxidant, preventing ROS-induced lung damage and rapid oxidation of ascorbate occurs during acute inflammation in acute lung injury [95]. In a mouse-model, the supplementation of vitamin C preserved lung barrier function and functionality of ion pumps in the alveolar epithelium [101] and decreased the lung pathology in an in vivo study of influence virus infected mice [102]. In rats, vitamin C attenuated lung injury caused by I/R [103]. 

A study conducted in 2016 found that vitamin C treatment of human bronchial epithelial cells attenuates particulate matter induced ROS damage, IL-6 expression and increased cell viability [104]. Vitamin C additionally attenuated smoking induced pulmonary emphysema and vascular remolding by reducing ROS induced protein oxidation [105]. In a study by Nathens et al. in 2002, the application of vitamin C decreased risk for pneumonia and ARDS with lower alveolar inflammation in a cohort of 270 mostly trauma patients [53]. Even though the results of this RCT did not reach statistical significance, they sparked further investigations on the subject. In the OMEGA study, Rice et al. supplemented antioxidant cocktails to ARDS patients and observed no benefit [106]. However, these cocktails contained many components and the 2 g/day vitamin C was only a minor component. In an RCT by Gadek et al., a combination of antioxidants, including vitamin C, decreased pulmonary inflammation and showed beneficial effects on gas exchange and requirement of mechanical ventilation in patients with ARDS [107].

#### 2.3.3. Vitamin C’s Influence on the Respiratory System in Cardiac Surgery Patients

Even if preclinical and clinical data seem promising, only very few studies addressed the effect of vitamin C on pulmonary dysfunction in cardiac surgery. To our knowledge, the duration of mechanical ventilation was the only outcome parameter measured in RCTs investigating this matter. Reduced intubation time after cardiac surgery was shown in a meta-analysis including 3 RCTs and 575 patients (mean difference: −2.41, 95% confidence interval −3.82/−0.98, *p* = 0.001). However, the heterogeneity of the included trials was high (*p* = 0.74) [63].

### 2.4. Renal System

#### 2.4.1. Renal Dysfunction after Cardiac Surgery

**Figure 5:**
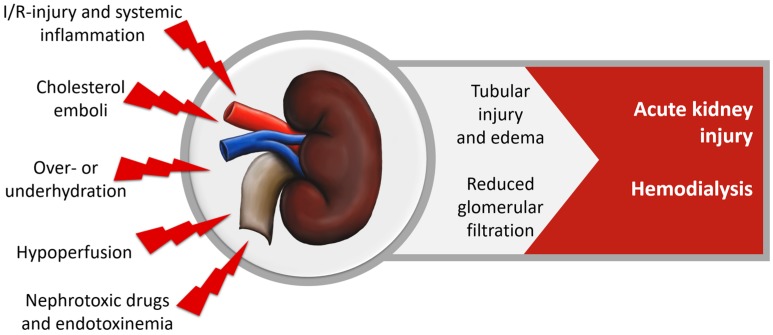
Renal dysfunction in cardiac surgery.

Acute kidney injury (AKI) is clinically one of the most significant organ dysfunction and occurs in about 28% of cardiac surgery patients [108], with 2–5% of patients requiring dialysis. Contributing factors are oxidative stress during renal I/R-injury, inflammation, hemolysis, cholesterol emboli, nephrotoxic drugs and toxins resulting in glomerular and tubular damage, reduced glomerular filtration rates and impaired creatinine clearance, as shown in Figure 5. AKI is strongly associated with the need for renal replacement therapy, increased hospital- and ICU-LOS, mortality and decreased long-term quality of life [3,108,109,110,111,112,113,114].

#### 2.4.2. Role of Vitamin C in the Renal System

The protective properties of vitamin C on the renal system are also attributed to its anti-oxidant capabilities. Vitamin C administration reduced serum creatinine levels in patients who experienced contrast-mediated nephropathy after coronary angiography [115]. These findings were supported by a meta-analysis including 1.536 patients in 9 RCTs in 2013 by Sadat et al., decreasing risk for AKI by 33% (risk ratio 0.672, confidence interval 0.466–0.969, *p* = 0.034) [116]. In contrast, excessive and long-term vitamin C consumption might lead to oxalate nephropathy. In a case report in 2012, Gurm et al. described a woman who consumed 3–6.5 g of vitamin C daily [117]. A similar case was reported in 2015: A 96-year-old woman was also diagnosed with oxalate nephropathy resulting from excessive vitamin C intake [118]. The tubular injuries are thereby caused by crystalline deposits of calcium oxalate, which might be metabolized from vitamin C. Therefore, the recurring formation of kidney stones, as well as chronic renal failure and hyperoxaluria are contraindications for a high-dose long-term vitamin C therapy, even though adverse effects seem unlikely in short-term administration [27,49]. In an RCT study including burn patients, decreased volume requirement for fluid resuscitation, as well as increased urine output were observed [55].

#### 2.4.3. Vitamin C’s Influence on the Renal System in Cardiac Surgery Patients

A pilot study by Antonic et al. in 2017 with 100 on-pump coronary artery bypass graft (CABG) surgery patients was not able to confirm the assumed benefits of vitamin C on renal function [119]. Potential causes for the insignificance of the results might be a rather low dosage and oral administration of vitamin C (2 × 1 g/day), as discussed in greater detail in Section 5.2. In any case, further research is warranted to investigate the effect of a high-dosage intravenous vitamin C application, to fully achieve the antioxidant and possibly nephroprotective effects.

### 2.5. Gastrointestinal System

#### 2.5.1. Gastrointestinal Dysfunction after Cardiac Surgery

**Figure 6:**
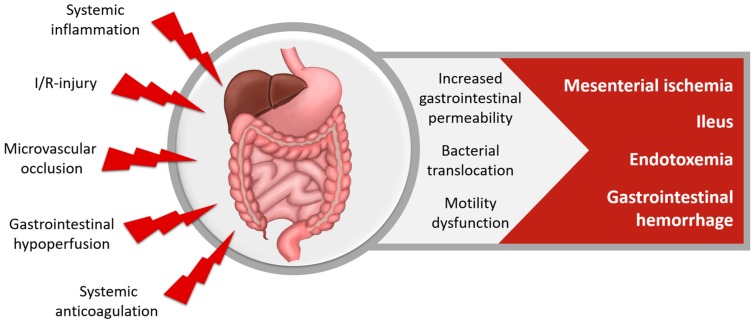
Gastrointestinal dysfunction after cardiac surgery.

Gastrointestinal (GI) complications (Figure 6) occur in 0.2–4% [120], while a postoperative gastrointestinal atony is observed in most cardiac surgery patients [121,122]. Inflammation and I/R-injury increase GI permeability and can lead to bacterial translocation and systemic endotoxemia. The most common GI complications are postoperative ileus and GI hemorrhage, while mesenteric ischemia and intestinal perforation are GI complications with the highest mortality. GI complications increase LOS and mortality [3,10,123,124,125].

#### 2.5.2. Role of Vitamin C in the Gastrointestinal System

The few available studies on the interaction of vitamin C with the GI system are derived from oncology. Vitamin C treatment might mitigate GI adverse effects associated with cancer treatment [126], where chemotherapy is often associated with damage to the mucous membrane. Al-Asmari et al. found attenuated toxicity of the antineoplastic drug 5 fluorouracil when vitamin C was administered, demonstrated by decreased activation of nuclear factor kappa-light-chain-enhancer of activated B cells (NF-κB) and cyclooxygenase-2 expression as well as lower infiltration of neutrophils [64]. The authors suggested that the observed benefits were due to the antioxidative effects of vitamin C. Similar findings were observed by Yamamoto et al. in 2010, who showed that vitamin C treatment attenuated the expression of apoptosis related genes as well as DNA damage in crypt cells caused by radiation [44].

#### 2.5.3. Vitamin C’s Influence on the Gastrointestinal System in Cardiac Surgery Patients

To our knowledge, no study of vitamin C in cardiac surgery reported beneficial or adverse effects on the GI system.

### 2.6. Coagulation System

#### 2.6.1. Coagulation Disorders after Cardiac Surgery

**Figure 7:**
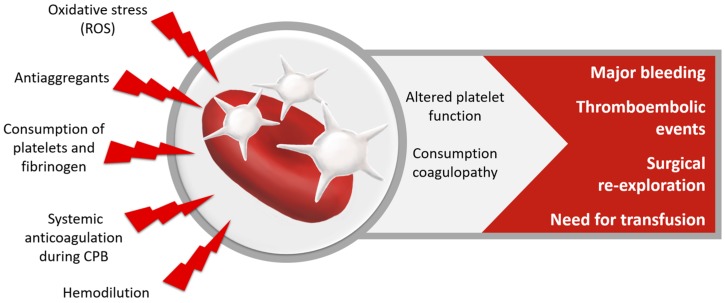
Coagulation disorders after cardiac surgery.

Coagulation disorders—both prothrombotic activity and coagulopathy—have deleterious effects on patient outcome (Figure 7). I/R induces the production of ROS by platelets and other vascular sources. ROS can alter platelet function and increase platelet aggregation and thrombus formation [127,128]. In a vicious circle, ROS-production and platelet-activation augment each other. Therefore, ROS may act prothrombotic. Additionally, reduced NO-responsiveness of the platelets might promote adhesion of the platelets to the endothelium, which is associated with increased cardiovascular morbidity in patients with acute coronary syndrome [127]. On the other hand, intra- and postoperative coagulopathy, commonly observed after cardiac surgery, lead to an increased need for transfusion of blood products and surgical re-exploration. The definition of bleeding is still debated [129], but mild bleeding occurs in almost one fifth and major bleeding in 3–12% of cardiac surgery patients [130]. A mean blood volume of 470 mL is lost during the first 12 h after cardiac surgery [131]. Contributing factors to coagulopathy are consumption and dilution of platelets and coagulation factors and heparinization during CPB, as well as effects of preoperative drugs and preexisting anemia and low fibrinogen-levels. The transfusion of the allogeneic blood products is associated with inflammation, transfusion-associated lung- and kidney injury and increases risk of stroke [66]. Overall, coagulopathy and major bleeding increase the risk of stroke, acute kidney injury, infections, surgical reoperation, LOS and mortality [130,131,132].

#### 2.6.2. Role of Vitamin C in the Coagulation System

Vitamin C has a tremendous impact on cellular and plasmatic hemostasis in the human body and has both pro- and anticoagulatory effects. The interaction between coagulation and vitamin C supplementation was already discussed in the early 1960s by Dayton and Weiner [133]. 

On a cellular level, antioxidants such as vitamin C may inhibit platelets by scavenging ROS, disrupting the vicious circle of ROS-platelet-activation, and restoring normal platelet function [127]. In healthy individuals, prostacyclin and NO prohibit platelet activation and prevent thrombosis. Vitamin C, however, inhibits the expression of iNOS in endothelial cells and nNOS in neurons and thereby lowers the plasmatic level of NO [43], hence acting pro-coagulatory. However, vitamin C also prevents microthrombus formation through inhibition of thrombin-induced and P-selectin mediated platelet aggregation and platelet-endothelial adhesion [43]. Even after the onset of microthrombus formation, ascorbate injection reverses capillary plugging and platelet-endothelial adhesion [43]. Vitamin C also inhibits the pH-dependent thrombin-induced release of plasminogen-activator-inhibitor-1 from platelets [43].

Plasmatic coagulation is influenced by vitamin C via several pathways. ROS and other stimuli activate NF-κB. The transcription factor NF-κB initiates the expression of cytokines and proteins involved in coagulation, such as tissue factor [134]. This suggests that coagulation via NF-κB can be affected by vitamin C [135,136]. Furthermore, vitamin C decreases tissue plasminogen activator and von Willebrand-factor, demonstrating an important link between inflammation, coagulation and vitamin C [136,137]. Vitamin C is also known to restore the capacity for endogenous, endothelium-dependent fibrinolysis in smokers [138].

On a systemic level, the influence of vitamin C on hemostasis might be dose-dependent. While depleted vitamin C levels are associated with gastrointestinal hemorrhage, especially in patients undergoing acetylsalicylate-treatment [139], in very high dosages (0.5–1 g/kg), vitamin C was found to promote the occurrence of thrombosis through pro-coagulant activation of erythrocytes in a rat model [140]. Vitamin C abolished coagulation abnormalities in septic mouse blood [101] and attenuated a sepsis-induced drop of thrombocytes in the systemic blood in septic patients [43].

#### 2.6.3. Vitamin C’s Influence on the Coagulation System in Cardiac Surgery Patients

To our knowledge, only two studies of vitamin C in cardiac surgery have addressed the issue of hemostasis. In one RCT from Sadeghpour et al. (*n* = 290), vitamin C reduced chest tube bleeding [141], while no difference was shown in another RCT [87]. Clearly, further research is needed to determine the influence of vitamin C on blood loss, need for transfusion and risk of thromboembolic events and to translate biochemical pathways into clinically relevant outcomes.

### 2.7. Immune System

#### 2.7.1. Immune Dysfunction after Cardiac Surgery

**Figure 8:**
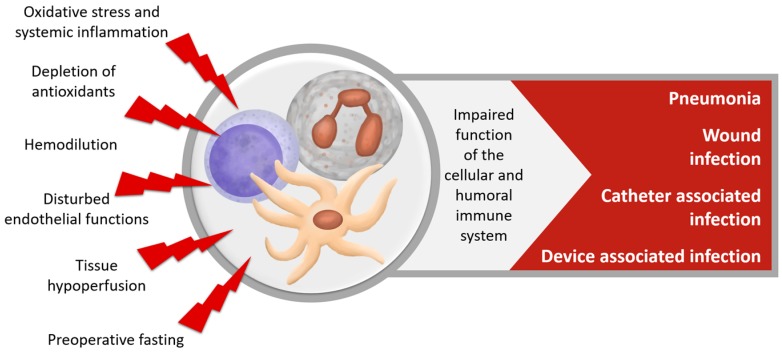
Dysfunction of the immune system after cardiac surgery.

After cardiac surgery, infections are the most common non-cardiac complication [142], (Figure 8). A quarter of all patients undergoing high-risk heart-surgery are diagnosed with a postoperative infection [143], and nearly 5% experience major infection. Pneumonia is the most frequent nosocomial infection in half of these cases. Surgical site infections and catheter- and device-associated infections each make up 25% of infections [143,144]. Major infections have a tremendous effect on subsequent survival and are associated with longer mechanical ventilation, ICU- and hospital stay and a higher morbidity and mortality up to five years after the operation [93,142,143,144,145,146,147,148,149,150,151,152].

#### 2.7.2. Role of Vitamin C in the Immune System

Infections are associated with and accompanied by an increase of oxidative stress. The increased ROS production during infection, and hypermetabolic vitamin C requirements are the reasons for the observed vitamin C reduction [38,153]. Evidence regarding increased vitamin C requirements in critical illness and after surgery will furthermore be discussed in Section 5.2.1.

Vitamin C is actively accumulated into the dermal cells and neutrophils via the sodium-dependent vitamin C transporters (SVCT). Neutrophils further increase their intracellular vitamin C concentration through uptake of DHA via glucose transporters (GLUT) and metabolization to ascorbate [38]. The accumulation of vitamin C in phagocytotic cells can enhance chemotaxis, phagocytosis, generation of ROS and microbial killing. Vitamin C is also necessary for apoptosis and the clearing of spent neutrophils from the infected site and enhances the proliferation and differentiation of B and T-cells, as well as antibody levels [38]. Vitamin C deficiency results in impaired immunity and thus, higher susceptibility for infections. 

Vitamin C supports endothelial barrier function against pathogens and promotes antioxidant scavenging activity of the skin. Vitamin C is a known inhibitor of bacterial growth, such as *S. aureus* and intestinal bacteria. One possible mechanism for the antibacterial function of vitamin C is the production of hydrogen peroxide during its oxidation [47]. Vitamin C also shortens time for wound healing through stimulation of proliferation, differentiation and migration of keratinocytes and fibroblasts, as well as through the stimulation of lipid synthesis [38]. Vitamin C enhances microbial killing through improved immune cells chemotaxis, motility and phagocytosis, and decreases necrosis through facilitation of apoptosis and clearance [38].

However, increased ROS production by the immune system is an important response to invasive pathogens. Therefore, suggesting that the radical-scavenging role of vitamin C is solely beneficial remains a matter of debate and is most likely dose-dependent. The systemic effect of vitamin C on bacterial and viral infections needs further research; current evidence demonstrates that vitamin C might prevent the development (or help ameliorate) of the clinical course of pneumonia [154,155]. Vitamin C deficiency was associated with increased inflammation as measured in CRP, and patients with septic shock were deficient in vitamin C in 40%, as observed in a study by Carr et al. [46].

#### 2.7.3. Vitamin C’s Influence on the Immune System in Cardiac Surgery Patients

Unfortunately, again, there is little knowledge about the influence of vitamin C on postoperative immune function and infections in cardiac surgery. Sadeghpour et al. reported a significant reduction in composite outcome “complications”, defined as death, infection, impairment in renal function and need for reoperation [141]. Neither the incidence of infection nor the influence of infection on the combined outcome parameter were reported in this study. Jouybar 2012 et al. [156] showed no difference in white blood count and inflammatory mediators using two bolus dosages of 3 g of vitamin C, 12–18 h before surgery and during CPB initiation.

## 3. Influence of Vitamin C on the Overall Clinical Outcome of Cardiac Surgery Patients

Considering the above-mentioned evidence and the data gained from meta-analyses and RCTs, as listed in Table 2 and Table 3, vitamin C may have positive effects on many vital functions and organ systems, which overall may have beneficial effects on patients’ short, mid and long-term outcomes.

- The overall effect is reflected by a reduced ICU-LOS in a meta-analysis of Geng et al. including 12 RCTs and 1584 patients [63] and Baker et al., including 11 RCTs and 1390 patients [89].
- Reduced hospital LOS was demonstrated in a systematic 2014 review including 5 RCTs [88], as well as the meta-analyses of Geng [63] and Baker [89] and Shi et al., including 13 trials involving 1956 patients [90]. However, in the meta-analysis by Hu et al. including 8 RCTs and 1060 patients, vitamin C application was not associated with reductions in ICU or hospital-LOS [59].
- Vitamin C might also reduce intubation time and postoperative complications as found by the meta-analyses of Hu and Shi [63,90].

Effects on LOS in un-blinded studies are subject to performance bias due to co-interventions or differentially applied policies on discharge. Additionally, none of the available RCTs included in these meta-analyses was adequately powered to detect an influence of vitamin C on overall clinical outcomes, such as on LOS or mortality, as discussed by Polymeropoulos et al. [60]. All meta-analyses found significant clinical and methodological heterogeneity of the included studies, limiting the strength of inferences. Therefore, there is an urgent need for additional large and well-designed clinical trials.

## 4. Vitamin C in Combination with other Antioxidant Therapies

Vitamin C has been combined with other antioxidant substances to minimize oxidative damage, as well as with anti-arrhythmic drugs such as beta-blockers and diltiazem to reduce the incidence of postoperative cardiac arrhythmia. In combination with beta-blockers, the incidences of AF and ICU-LOS were significantly reduced compared to CABG-patients who only received beta-blocker pre-surgery [87].

Vitamin C also regenerates α-Tocopherol (vitamin E); therefore, a combination therapy might offer more benefits compared to a monotherapy [19]. A combination of vitamins C and E significantly reduced 28-day mortality and duration of mechanical ventilation in ICU patients, in a study by Crimi et al. [170]. Howe et al. observed a reduction of mechanical ventilation and a trend towards reduced all-cause mortality and ICU-LOS in critically ill patients [171]. In cardiac surgery patients, the combined vitamin C and E therapy lowered oxidative stress, as demonstrated by lower lipid oxidation and lysosomal enzyme activity [172], improved function of the pulmonary vessels [173] and seemed to have an anti-inflammatory effect, as measured in lower CRP levels in a study by Gunes et al. [174]; see also Table 3.

## 5. Practical Approach to Vitamin C Supplementation

### 5.1. Risks and Side Effects

As demonstrated above, many studies have supplemented vitamin C, but significant adverse effects on patients in short term use have not yet been reported. This is true for low, as well as for dosages of 200 mg/kg/day and up to extremely high dosages of 1500 mg/kg three times a week in cancer patients [49]. Possible adverse effects are related to dosage, enteral route, and duration of vitamin C supplementation and include:

- Diarrhea and abdominal bloating [27]
- False negative tests for gastrointestinal occult bleeding [27]
- Aggravation of iron overload in patients with hemochromatosis or other diseases requiring frequent blood transfusions, such as thalassemia major and sideroblastic anemia [27]
- Possible adverse pro-oxidative effect in large dosages in case of iron overload [49]
- Possible hyperuricosuria [27]
- Formation of kidney stones through precipitation of calcium oxalate, especially in patients with chronic renal failure, hyperoxaluria and recurring formation of kidney stones [27,49]
- Hemolysis in patients with hereditary glucose-6-phosphate dehydrogenase (G6DP) deficiency, when administered in high dosages of >4 g/day [27]
- False-high measurements of blood glucose in hand-held devices [179,180].

Importantly, vitamin C can act not only as antioxidant, but also as pro-oxidant in the presence of redox-active transition metal ions [181]. In theory, the reduction of metal ions (e.g., iron or copper) by vitamin C in vitro can result in the formation of highly reactive hydroxyl radicals via reaction of the reduced metal ions with hydrogen peroxide, which is known as Fenton chemistry. Yet, this reaction requires the availability of free, redox-active metal ions and a low ratio of vitamin C to metal ion, which is unlikely to occur in human biology under normal circumstances [181,182,183]. Vitamin C might act as prodrug to hydrogen peroxide even without metal chelators when administered in pharmacologic concentrations but led to no hydrogen peroxide accumulation in the blood vivo [184]. In this context, a few studies [185] have suggested a pro-oxidative effect of vitamin C, portrayed as an underlying reason for negative effects, although none of these ever confirmed a causative relation.

### 5.2. Application Strategies

#### 5.2.1. Dosing

Current literature does not support a specific vitamin C dosing strategy in cardiac surgery, in the absence of a definitive trial. The dose typically administered by parenteral and enteral nutrition is 200 mg/day, which is recommended for the healthy population. In a study by Carr et al., standard enteral or parenteral nutritional therapy with a mean of 125 mg/day did not prevent hypovitaminosis C in critically ill patients [46]. Even after less invasive and elective surgery, such as maxilla-facial surgery, higher dosages (500–2000 mg/day, mean 1150 mg/day) were required to increase plasma vitamin C levels and compensate for the observed loss [47,186,187]. In patients experiencing significant inflammation and oxidative stress, such as trauma, burn, sepsis and cardiac surgery patients, the vitamin C requirement seems to increase dramatically. A dosage of 3–4 g/day parenterally seems necessary to normalize the vitamin C plasma levels in patients with burns or sepsis [49] or critically ill trauma patients [48]. Probable causes for this high demand are higher consumption due to the antioxidant capacity of vitamin C, as well as increased renal clearance during vitamin C substitution.

Fowler et al. recently published a phase 1 clinical trial, suggesting that 200 mg/kg/day yields higher plasma levels of vitamin C and more favorable Sequential Organ Failure Assessment (SOFA) scores compared to 50 mg/kg/day in severely septic patients [51]. A very high dosage of 66 mg/kg/h for the first 24 h was used in the study by Tanaka et al. in burn patients, which led to reduced fluid demand and increased urine production [54,55].

In cardiac surgery patients, the dosing regimen used in the previously mentioned studies are extremely heterogenous. Most studies use a single dose of 2 g once prior to surgery. Postoperatively, a very small dosage of less than 1 g/day was administered in many studies [141,157,159,162,163,165,167]. However, single-dosages as high as 150 mg/kg [86] or 250 mg/kg have also been applied [158]. To our knowledge, there is no dose-finding study on cardiac surgery patients yet.

#### 5.2.2. Timing

Oxidative damage is highest minutes after reperfusion; hence, early administration may be optimal. Logically, preoperative administration might refuel the body’s antioxidant capabilities, preparing for CPB. Application of a dosage before the removal of the aortic cross-clamp and reperfusion might achieve the minimal ROS-scavenging plasma-levels of 1–10 mmol/L [49].

In one study, the cardiac index was significantly higher in the first 6 h after the operation in patients receiving a mega-dose of 125 mg/kg, suggesting that the effect of vitamin C might wear off after that period of time [86]. Ruemelin et al. showed a rapid decrease in plasma concentration after the end of the infusion [187]. In a study by Tanaka [55], serum levels of vitamin C increased quickly under continuous infusion, remained elevated until 12 h after infusion and decreased rapidly.

To our knowledge, no trial studied the duration of vitamin C administration in cardiac surgery patients, or even in other critically ill patients. In cardiac surgery, the greatest trauma is related to the surgery itself, as well as to ischemia/reperfusion after cardiopulmonary bypass. The most relevant oxidative stress and inflammation is expected within the first hoursand days after surgery. Bjugstadt et al. demonstrated that oxidative stress remained significant, even days after injury, while the extent of oxidative stress on the fourth day after trauma was the most relevant for patients’ outcomes [188,189]. Therefore, the most reasonable duration for substitution might be 96 h and is also used by Fowler et al. ([51] and NCT02106975).

#### 5.2.3. Mode of Administration

The route of vitamin C administration may be of pivotal importance, but studies comparing the oral versus intravenous application in patients are still missing.

Studies showed that serum vitamin C cannot be raised to physiological levels after surgery, if administered orally, even if the highest tolerated dosage is administered enterally Intravenous application of vitamin C results in much higher circulating ascorbate concentrations [46], while the effects of these supra-physiological vitamin C concentrations have yet to be studied. On the other hand, even an oral application of vitamin C was shown to be beneficial in RCTs by Sadeghpour [141], and Dehghani [165].

Positive effects of high doses of vitamin C (if administered either orally or by infusion) on arterial vasodilation have been reported (review in detail in [28]). Although comprehensive studies, evaluating carefully the potential differences between oral and intravenous vitamin C, are missing, it is suggested that intravenous application provides advantages due to higher bioavailability and faster action time in the vascular system. When vitamin C is supplemented parenterally, supraphysiological dosages can safely be administered and the antioxidant effects of vitamin C may be increased [49]. 

Another issue is the application strategy of the vitamin. One possibility to counteract rapid metabolic clearance and drop of plasma vitamin C levels would be continuous infusion, which is feasible and effective under UV-protection [55]. However, vitamin C’s lability allows for degradation of the vitamin before it enters the patient. Another option might be frequent bolus dosing, as used in a trial by Fowler et al. [51]. 

#### 5.2.4. Monitoring

As outlined before, vitamin C can be measured in its oxidized form DHA. When monitoring DHA in blood samples, it has to be kept in mind that ascorbic acid is sensitive to oxidation and degradation during blood sampling, handling, storage and analysis. Therefore, the handling, storage and following shipment to reference laboratories may be problematic [190]. Factors influencing the stability of DHA in whole blood and serum are temperature, light-exposure, pH, contamination with copper or iron and anticoagulant of the blood sample [26,191], as well as dissolved oxygen, solvent, ionic strength, trace metals and oxidizing enzymes. In a refrigerator at 4 °C, the degradation of vitamin C within 24 h is 1.8% in serum tubes and 7.2% in plasma tubes [192].

Therefore, blood samples should be drawn immediately, stored in crushed ice in a light protected box, and be delivered within 2 h for reliable vitamin C measurements [193]. When whole blood is immediately centrifuged, acidified and stored at −70 °C, ascorbic acid degrades very slowly and can be analyzed for at least 6 years. However, due to different degradation rates depending on the acid and anticoagulant used in sampling tubes, a quick analysis seems preferable [26,194]. High performance liquid chromatography (HPLC) with electrochemical detection is the current gold standard of vitamin C measurement, which usually requires the stabilization of vitamin C through acid or alcohol precipitation, usually combined with a metal chelator [191]. Robitaille and Hoffer showed that the simpler UV light detection is equivalent to electrochemical detection [190]. A recent study by Pullar et al. demonstrated a good stability of DHA for up to a year at −80 °C, both as plasma, as well as in extracts with perchloric acid (PCA) containing 100 µmol/l of the metal chelator diethylenetriaminepentaacetic acid (DTPA) extracts, with a loss of 8% in 12 months [191].

Considering these influencing factors, the measurement of vitamin C is elaborate and costly and therefore, not readily accessible in the ICU.

## 6. Discussion and Future Directions

The ways in which vitamin C attenuates inflammation and oxidative damage and its clinical application is a current topic of interest. Preclinical as well as preliminary clinical studies have demonstrated the beneficial effects of vitamin C on organ function during inflammation and oxidative stress.

No serious adverse events have been reported in any of the cited studies, highlighting the safety of this pharmaco-nutrient. However, despite the outlined promising effects, only a few adequately designed clinical trials have been conducted to provide robust evidence about vitamin C’s influence on organ dysfunction. One cause might be the variance of the supplementation strategy (dose and timing) used in the existing studies. Besides, the overall importance of existing RCTs is weakened by the heterogeneity in outcome measuring and reporting, which hinders comparison of interventions. This problem is encountered by so-called core outcome sets. These core outcome sets include minimal outcome parameters, which should be measured and reported in clinical trials of a specific area. Their use ameliorates the comparison of trials’ results and their combination, if appropriate [195]. In the future, a specific core outcome set should be developed for trials investigating the effect of (pharmaco-)nutrition in cardiac surgery. Larger and adequately designed clinical studies are urgently needed, before any recommendation regarding the application of vitamin C in cardiac surgery can be made.

Any conclusive evidence of the benefits of vitamin C in cardiac surgery patients would lead to rapid implementation of this promising therapy for four reasons: (1) The overall safety profile of vitamin C may enable a broad use; (2) the feasibility of vitamin C administration without any dose adjustments; (3) familiarity for clinicians and patients as a therapy for cancer and in some burn units; (4) low costs to produce and administer.

## Figures and Tables

**Table 1:** Summary of vitamin C’s influence on organ systems.

Organ System	Influence of Vitamin C
Nervous system	Elevated levels protect neurons from oxidative damage [49,57]
	Reduces the infarct volume after ischemia [58]
Cardiovascular System	Attenuates myocardial damage and improves myocardial stunning [49]
	Reduces vasopressor demand [52]
	Reduces rate of atrial fibrillation [59,60]
	Improves endothelial function [61,62]
Respiratory System	Reduces intubation time [63]
	Decreases risk of pneumonia and alveolar inflammation [53]
Renal System	Reduces fluid demand and increases urine production [54,55]
Gastrointestinal System	Attenuates drug toxicity, decreases inflammatory reaction [64]
	Lowers infiltration of neutrophils [64]
	Reduces the expression of apoptosis related genes [44]
Coagulation System	Restores platelet function and decreases capillary plugging [43]
	Attenuates a sepsis-induced drop of thrombocytes [43]
Immune System	Inhibits bacterial growth [47], enhances microbial killing [38]
	Supports endothelial barrier function and promotes antioxidant scavenging [38]

**Table 2:** RCTs investigating the effects of vitamin C (Vit C) in cardiac surgery.

Author and Year	Patients	Dosage of Vitamin C	p.o./i.v.	Results
Knodell 1981 [157]	175+ hepatitis	Preop: 4 × 800 mg/day for 2 days	p.o.	Elevations of plasma vitamin C, no influence on the hepatitis
		Postop: 4 × 800 mg/day for 2 weeks		
Li 1990 [158]	20	Preop: 250 mg/kg before the start of extracorporeal circulation	N.A.	Sign. reduction in lipid peroxidation
Dingchao 1994 [86]	85 CPB	125 mg/kg 30 min before surgery and at the end of CPB	i.v.	Decreased CK/CKMB, LDH, & rate of defibrillation, ICU- and hospital LOS, improved CI
Carnes 2001 [159]	86 CABG	Preop: 1 × 2 g the night before	N.A.	Lower rate of AF
		Postop: 2 × 0.5 g/day for 5 days		
Demirag 2001 [160]	30 elective	Group 1: 2 × 50 mg/kg vitamin C at induction and end of CPB	i.v.	Prevention of lipid peroxidation no difference in myocardial I/R-injury
		Group 2: vitamin C + diltiazem: bolus and 2 µg/kg/min until end of CPB		
Eslami 2007 [161]	100 CABG	Preop: 1 × 2 g night before	p.o.	Lower rate of AF
		Postop: 2 × 1 g/day for 5 days		
Colby 2011 [162]	24 CABG and/or valve	Preop: 1 × 2 g night before	p.o.	No difference in CRP, WBC, fibrinogen, Trend: decreased AF, hospital- and ICU-LOS
		Postop: 2 × 0.5 g/day for 4 days		
Papoulidis 2011 [163]	170 CABG	Preop: 1 × 2 g 3 h prior to surgery	i.v.	Sign. lower rate of AF, hospital- and ICU-LOS
		Postop: 2 × 0.5 mg/day for 5 days		
Bjordahl 2012 [164]	185 CABG	Preop: 1 × 2 g night before surgery	p.o.	No difference in postoperative complications, mortality or AF
		Postop: 2 × 1 g/day for 5 days		
Jouybar 2012 [156]	40 CABG	Preop: 2 × 3 g 12–18 h before surgery and during CPB initiation	i.v.	No difference in inflammatory cytokines, hemodynamics, blood gases, urea nitrogen, creatinine, WBC, platelet counts & outcomes
Dehghani 2014 [165]	100 CABG	Preop: 1 × 2 g	p.o.	Sign. lower rate of AF, hospital- and ICU-LOS
		Postop: 2 × 0.5 g/day for 5 days		
Ebade 2014 [166]	40	Preop: 1 × 2 g	i.v.	Lower incidence of AF
		Postop: 1 × 1 g 12 h after surgery, 3 × 1 g for 6 days after surgery		Shortened ICU- and hospital-LOS
Sama-dikhah 2014 [167]	120 CABG	Preop: 1 × 2 g	p.o.	Sign. lower rate of AF
		Postop: 1 × 1 g/day for 5 days		
		Plus atorvastatin 40 mg		
Sadegh-pour 2015 [141]	290 CABG, valve	Preop: 1 × 2 g before surgery	Preop: i.v. Postop: p.o.	Sign. reductions in AF, hospital-LOS, intubation time, complications (death, renal function, infection) and drainage, unchanged ICU-LOS
		Postop: 1 × 1 g/day for 4 days		
Das 2016 [168]	70 elective low risk CABG	Preop: 2 × 0.5 g for 7 days prior to surgery	p.o.	Lower vasopressors-demand, no difference in time to extubation, ICU- and hospital-LOS, mortality or complications
Antonic 2016 [169]	105 CABG	Preop: 2 × 2 g: 24 and 2 h before surgery	i.v.	Trend: decreased rate of AF, no difference in complications
		Postop: 2 × 1 g/day for 4 days		
Antonic 2017 [119]	100 CABG	Preop: 2 × 2 g: 24 and 2 h	i.v.	No sign. protective effect of ascorbic acid on the incidence of postoperative AKI
		Postop: 2 × 1 g/day for 5 days		

CPB = cardiopulmonary bypass, CABG = coronary artery bypass graft, p.o. = per os, i.v. = intravenous, sign. = significantly, N.A. = not available, WBC = white blood count, preop = before surgery, postop = after surgery, LDH = lactate dehydrogenase.

**Table 3:** RCTs investigating antioxidant cocktails in cardiac surgery.

Author and Year	*N*	Treatment	Outcomes
Barta 1991 [172]	20	Preop: 2000 IU Vit E: 12 h before surgery; 2 g vitamin C in the morning on the day of surgery	Inhibition of the decrease of catalase Lower lipid oxidation and lysosomal enzymes in intervention group
Westhuyzen 1997 [175]	76	Preoperative (7–10 days): 1 g vitamin C and 750 IU Vit E	Supplementation of the vitamins prevented depletion, but provided no clinical advantage
Angdin 2003 [173]	22	Preop: 900 mg Vit E for 10–14 days plus 1 × 2 g vitamin C and 600 mg allopurinol the evening before surgery, and acetylcysteine during surgery	Reduction of pulmonary vascular endothelial dysfunction in the group treated with ntioxidants
Castillo 2011 [176]	95	Preop: for 7 days *n*-3 PUFA 2 g/day Plus, for 2 days preop until discharge vitamin C 1 g/day and Vit E 400 IU/day	Decrease in oxidative stress-related biomarkers in atrial tissue
Gunes 2012 [174]	59	Preop: vitamin C 500 mg and Vit E 300 mg Postop: vitamin C 500 mg/day and Vit E 300 mg/day for 4 days	Significant reduction of CRP
Rodrigo 2013 [177]	203	Preop: 1 g/day vitamin C plus PUFA and Vit E for 2 days preop until discharge	Decrease in oxidative stress-related biomarkers in atrial tissue
Stanger 2014 [178]	75	4 subgroups: control, vitamins, *n*-3 PUFAs, and a combination of vitamins and *n*-3 PUFAs	Attenuation of postop oxidative stress, Oxidative stress associated with consumption of antioxidants and onset of AF
		Vitamin group: 500 mg vitamin C + 45 IE Vit E 30 min before reperfusion, postop and 120 min after reperfusion	
Rezk 2017 [87]	100	3 days preoperatively	Significantly lower incidence in vitamin C group, ICU-LOS, need for inotropes and mechanical ventilation
		Group 1: β-blocker: 5 mg bisoprolol and 2 g/day vitamin C	
		Group 2: β-blocker only	

Vit E = vitamin E.

## Abbreviations

AF	Atrial Fibrillation
AKI	Acute Kidney Injury
CABG	Coronary Artery Bypass Graft
CPB	Cardiopulmonary Bypass
CRP	C-reactive Protein
DHA	Dehydroascorbate
GI	Gastrointestinal
ICU	Intensive Care Unit
iNOS	Inducible Nitric Oxide Synthetase
I/R	Ischemia/Reperfusion
i.v.	Intravenous
LOS	Length of Stay
N.A.	Not Available
NFκB	Nuclear Factor kappa-light-chain enhancer of activated B cells
nNOS	Neuronal Nitric Oxide Synthetase
NO	Nitric Oxide
PCA	Perchloric Acid
p.o.	Per Os
Postop	Before Surgery
Preop	After Surgery
RCT	Randomized Controlled Trial
ROS	Reactive Oxygen Species
Sign.	Significantly
SIRS	Systemic Inflammatory Response Syndrome
SOFA	Sequential Organ Failure Assessment
SVCT2	Sodium-dependent Vitamin C Transporter-2
TNFα	Tumor Necrosis Factor α
Vit C	Vitamin C
Vit E	Vitamin E/α-Tocopherol
WBC	White Blood Count
